# Gold-catalyzed ethylene cyclopropanation

**DOI:** 10.3762/bjoc.15.7

**Published:** 2019-01-07

**Authors:** Silvia G Rull, Andrea Olmos, Pedro J Pérez

**Affiliations:** 1 Laboratorio de Catálisis Homogénea, Unidad Asociada al CSIC, CIQSO-Centro de Investigación en Química Sostenible and Departamento de Química, Universidad de Huelva, Campus de El Carmen 21007 Huelva, Spain

**Keywords:** carbene transfer, cyclopropane, cyclopropylcarboxylate, ethylene cyclopropanation, ethyl diazoacetate, gold catalysis

## Abstract

Ethylene can be directly converted into ethyl 1-cyclopropylcarboxylate upon reaction with ethyl diazoacetate (N_2_CHCO_2_Et, EDA) in the presence of catalytic amounts of IPrAuCl/NaBAr^F^_4_ (IPr = 1,3-bis(2,6-diisopropylphenyl)imidazole-2-ylidene; BAr^F^_4_ = tetrakis(3,5-bis(trifluoromethyl)phenyl)borate).

## Introduction

**Scheme 1:**
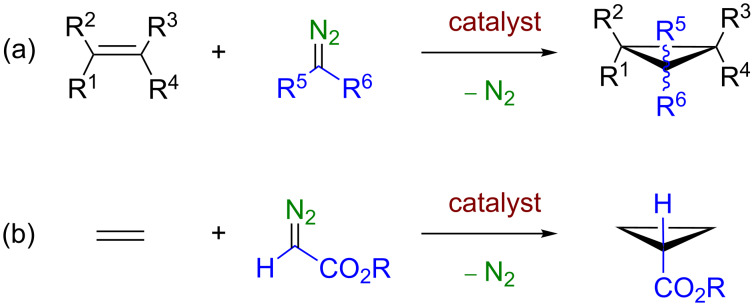
(a) General metal-catalyzed olefin cyclopropanation reaction with diazo compounds. (b) The ethylene cyclopropanation with diazoacetates leads to cyclopropanecarboxylates.

**Scheme 2:**
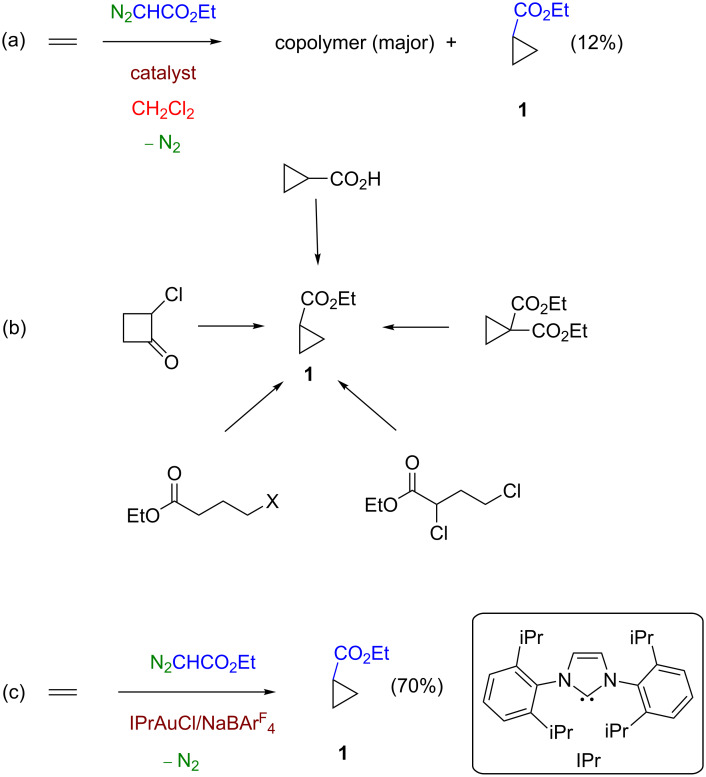
Routes toward ethyl cyclopropanecarboxylate (**1**). (a) Ethylene cyclopropanation described by De Bruin and co-workers as secondary reaction. (b) Stoichiometric transformations. (c) Gold-catalyzed ethylene cyclopropanation described in this work.

Nowadays the olefin cyclopropanation through metal-catalyzed carbene transfer starting from diazo compounds to give olefins constitutes a well-developed tool (Scheme 1a), with an exquisite control of chemo-, enantio- and/or diastereoselectivity being achieved [1–2]. Previous developments have involved a large number of C=C-containing substrates but, to date, the methodology has not been yet employed with the simplest olefin, ethylene, for synthetic purposes [3]. Since diazo compounds bearing a carboxylate substituent are the most commonly employed carbene precursors toward olefin cyclopropanation, their use with ethylene leads to cyclopropane (Scheme 1b). De Bruin and co-workers have described [4] such product in a minor, secondary reaction (yields <12%) while studying the copolymerization of ethylene and ethyl diazoacetate with rhodium-based catalysts (Scheme 2a).

Ethyl cyclopropanecarboxylate has been prepared in several ways, alternative to the direct carbene addition to ethylene (Scheme 2b): ring contraction of 2-halocyclobutanone [5], cyclization of alkyl 4-halobutanoates [6], electroreductive dehalogenation [7] and decarboxylation of diethyl 1,1-cyclopropyldicarboxylate [8]. Other methods include the transesterification of other alkyl cyclopropanecarboxylates [9] and the esterification with ethanol of the cyclopropanecarboxylic acid [10]. This product finds applications as lubricant additives [11], alkylating reagent in the Friedel–Crafts synthesis of indanones [12] or as synthon toward the introduction of cyclopropyl moieties in compounds with medicinal or biological interest [13–14].

In view of the lack of examples of direct conversions of ethylene into cyclopropanecarboxylates, and given our experience with group 11 metal-based catalysts for carbene-transfer reactions from diazoacetates [15–16], we have investigated this transformation and found that the gold complex IPrAuCl (IPr = 1,3-bis(2,6-diisopropylphenyl)imidazole-2-ylidene) along with one equivalent of NaBAr^F^_4_ (BAr^F^_4_ = tetrakis(3,5-bis(trifluoromethyl)phenyl)borate) catalyzes the ethylene cyclopropanation with ethyl diazoacetate as the carbene precursor, under mild conditions, with moderate yields (ca. 70%, EDA-based).

## Results and Discussion

Diazo compounds N_2_=CRR’ usually react with metal complexes of groups 8–11 with formation of electrophilic metal–carbene intermediates L*_n_*M=CRR’ [1–2] that further react with nucleophiles such as olefins en route to cyclopropanes. However, these intermediates can also react with another molecule of the diazo reagent promoting the formation of olefins RR’C=CRR’ [17]. This side reaction frequently is avoided upon maintaining a low diazo compound/catalyst ratio, employing slow addition devices to incorporate a solution of the diazo reagent into the reaction mixture containing the olefin and the catalyst. Unfortunately, the use of ethylene as the olefin requires a pressure vessel and thus the diazo reagent must be added in one portion before pressurizing the system. This fact constitutes the main drawback when working with this alkene, and a highly chemoselective catalyst toward cyclopropanation over carbene dimerization is needed to enhance the former transformation.

In a first array of experiments, we tested several group 11 metal-based catalysts that have been described in our group for the catalytic transfer of the CHCO_2_Et group from ethyl diazoacetate (N_2_=CHCO_2_Et, EDA), as well as a rhodium-based catalyst. The experimental procedure started upon placing the catalyst (0.02 mmol) into a Fischer–Porter vessel and addition of an EDA solution in 10 mL of dichloromethane via cannula under ethylene atmosphere, and then it was pressurized to 8 bar with the same gas. The mixture was stirred for 14 h and then investigated by GC (see the experimental section). The results are collected in Table 1. The complex Tp^Ms^Cu(thf), previously described as an excellent catalyst for olefin cyclopropanation [18], led to negative results, since only the olefins **2** (mixtures of diethyl fumarate and maleate) were detected at the end of the reaction. The second copper-based catalyst tested Tp^(CF3)2,Br^Cu(thf) [19] gave some of the desired product **1** (Table 1, entry 2), albeit in low yield (15%). Olefins **2** were also formed, although mass balance was not verified by GC studies. When the crude was analyzed by NMR, broad signals characteristic of polymeric materials were observed. Given that our goal was the development of a catalytic route for cyclopropane **1**, we have not invested efforts in the characterization of such materials.

**Table 1:** Catalyst screening for the reaction of ethylene and ethyl diazoacetate.^a^

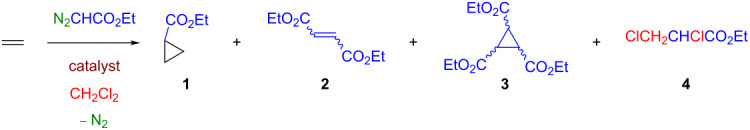						

Entry	Catalyst	% **1**	% **2**	% **3**	% **4**	% EDA

1	Tp^Ms^Cu(thf)	0	>95	nd	nd	nd
2	Tp^(CF3)2,Br^Cu(thf)	15	15	nd	nd	nd^b^
3	Tp^(CF3)2,Br^Ag(thf)	5	nd	nd	>75	13
4	IPrAuCl	0	4	0	0	40^b^
5	IPrAuCl + AgOTf	2	2	9	nd	80
6	IPrAuCl + AgSbF_6_	25	0	nd	>70	nd
7	IPrAuCl + NaBAr^F^_4_	62	2	nd	nd	36
8	Rh_2_(CF_3_COO)_4_	41	14	nd	nd	nd^b^

^a^Reaction carried out with 0.02 mmol of catalyst, 0.2 mmol of EDA except with the rhodium catalyst, which was run with 0.4 mmol of EDA. Ethylene pressure = 8 bar. Solvent: 10 mL of dichloromethane. Room temperature. Product determination and quantification by GC with calibration curves, yields based on initial EDA. nd = not detected. ^b^NMR studies show broad signals characteristic of polymeric materials accounting for 100% of initial EDA.

The silver complex Tp^(CF3)2,Br^Ag(thf) [19] (Table 1, entry 3) was not effective toward the aforementioned target, since only 5% of **1** was formed. In this case, the product derived from the insertion of the carbene CHCO_2_Et group into the C–Cl of the solvent was the major one, accordingly with previous work from this and other laboratories using silver-based catalysts [20–21]. Therefore, we moved onto gold-based catalysts that had already been validated for EDA decomposition and carbene-transfer reactions [22–23]. Neutral IPrAuCl was not effective (Table 1, entry 4), assessing the need of a cationic, halide-free gold species toward that end. The choice of the halide scavenger is not innocent: on the other hand, it is key for the success of this search. Thus, addition of one equiv of AgOTf with respect to the gold complex resulted in low consumption of EDA, and minor amounts of **1**, olefins **2** and cyclopropane **3** derived from carbene addition to **2** were detected. The use of AgSbF_6_ led to different results: the yield into desired **1** increased up to 25% but the functionalization of the solvent (**4**) constituted the main transformation. This is probably the effect of the silver in the reaction medium, since simple silver salts promote such reaction. The use of NaBAr^F^_4_ delivered ethyl cyclopropanecarboxylate (**1**) in 62% yield, with only 2% of olefins **2** as byproducts, the remaining 36% of initial EDA appearing unreacted at the end of the 14 h period. When the dirhodium complex Rh_2_(CF_3_COO)_4_ was submitted to the same experimental conditions, compound **1** was detected in 41% yield, along with 14% of olefins **2**. Again, the analysis of the crude mixture through NMR revealed the presence of polymeric material.

**Figure 1:**
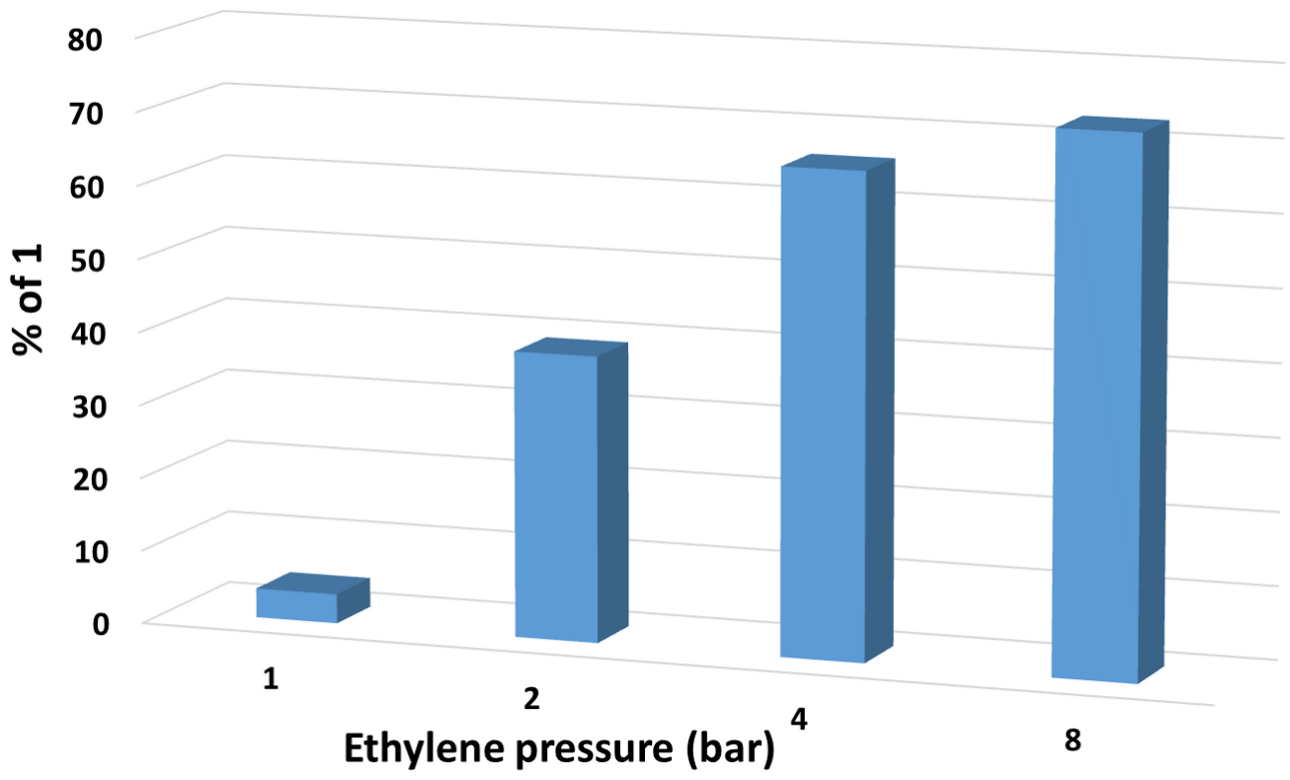
Effect of the pressure of ethylene on the yields of ethyl cyclopropanecarboxylate in the reaction of ethylene and EDA catalyzed by IPrAuCl/NaBAr^F^_4._

Once the IPrAuCl/NaBAr^F^_4_ was identified as the best choice toward the catalytic formation of **1**, several reaction conditions were explored. As shown in Table 2, and plotted in Figure 1, four experiments carried out at 1, 2, 4 and 8 bar of ethylene (Table 2, entries 1–4) revealed that the latter was the optimal value. This is clearly the effect of the need of a high concentration of the olefin in the reaction mixture, which is proportional to the partial pressure above the solution. Also, the use of a 5 mL volume of the solvent instead of 10 mL not only allowed to increase the yield up to 73% but also to induce complete consumption of ethyl diazoacetate. The effect of the temperature when moving from ambient (Table 2, entry 4) to 40 °C (Table 2, entry 5) was negligible, albeit in the former the cyclopropane **3** was observed. Again, some polymeric material was detected by NMR spectroscopy. It is worth mentioning that when diethyl diazomalonate or ethyl 2-phenyldiazoacetate were employed as the carbene precursor, no cyclopropanes were detected, only olefins formed from carbene dimerization as well as unreacted diazo compounds were identified at the end of the reaction.

**Table 2:** Catalytic activity of IPrAuCl/NaBAr^F^_4_ in the reaction of ethylene and ethyl diazoacetate.^a^

Entry	P(C_2_H_4_) (bar)	V(CH_2_Cl_2_) (mL)	% **1**	% **2**	% **3**	% EDA

1	1	5	5	>90	nd	nd
2	2	5	39	2	35	12
3	4	5	66	0	33	nd
4	8	5	73	0	15	nd^b^
5^c^	8	5	70	0	0	nd^b^
6	8	10	62	2	0	36

^a^Conditions and product numbering as described in Table 1. nd = not detected. ^b^NMR studies show broad signals characteristic of polymeric materials accounting for 100% of initial EDA. ^c^Reaction performed at 40 °C.

## Conclusion

We have found that the complex IPrAuCl in the presence of one equivalent of NaBAr^F^_4_ catalyzes the reaction of ethyl diazoacetate and ethylene, at room temperature, leading to ethyl cyclopropanecarboxylate with yields of ca. 70% (EDA-based). This is the first example of a direct cyclopropanation of ethylene by this methodology with significant conversions.

## Experimental

### General methods

All preparations and manipulations were carried out under an oxygen-free nitrogen atmosphere using conventional Schlenk techniques. Solvents were rigorously dried prior to use. The substrates as well as compound **1** (for calibration curves) were purchased from Aldrich. The catalysts were prepared according to literature procedures, as well as NaBAr^F^_4_ [24]. NMR spectra were performed on Agilent 400 MR and 500 DD2. GC data were collected with an Agilent 7820A equipped with an FID detector and an Agilent HP-5 column of 30 m × 320 μm × 0.25 μm. Method: 50 °C × 1.5 min, 10 °C/min, 250 °C × 25 min.

### Catalytic experiments

**Ethylene cyclopropanation with EDA.** A 175 mL high pressure Fischer–Porter vessel equipped with a manometer and a valve was charged with 0.02 mmol of IPrAuCl (24 mg) and 0.02 mmol of NaBAr^F^_4_ (17 mg). The vessel was deoxygenated and filled with ethylene. A solution of 0.2 mmol of EDA (24 μL) in 5 mL anhydrous dichloromethane was added via cannula and the ethylene pressure was increased up to 8 bar. The reaction mixture was stirred for 14 hours and the pressure was released.

**Reaction mixture analysis.** The crude reaction mixture was diluted to 10 mL and directly analyzed by GC. The amounts of ethyl cyclopropanecarboxylate (**1**), EDA, diethyl maleate and diethyl fumarate (**2**) were determined using calibration curves ranging from 2 mM to 20 mM, previously prepared using commercially available products. Retention times of products: **1**, 5.78 min; EDA, 5.90 min; diethyl maleate, 10.73 min; diethyl fumarate, 10.93 min. To determine the quantity of triethyl 1,2,3-cyclopropanetriscarboxylate (**3**) formed, the solution was evaporated and analyzed by NMR using CDCl_3_ as solvent and ethyl chloroacetate as internal standard.
